# Exploring the factors influencing evidence-based approaches to advanced chronic kidney disease: a qualitative study involving nurses and physicians

**DOI:** 10.1186/s12875-024-02418-0

**Published:** 2024-05-21

**Authors:** Elena Pintado-Outumuro, Victoria Morin-Fraile, Betlem Salvador-González, Llúcia Benito, Maricel Julve-Ibáñez, M.-Pilar Sancho-Campos, Carolina Alves-Tafur, Iris Lumillo-Gutiérrez

**Affiliations:** 1 Primary Care Center El Pla. Servei d’Atenció Primària Baix Llobregat Centre, Atenció Primària Metropolitana Sud, Institut Català de la Salut, Sant Feliu de Llobregat, Barcelona, 08980 Spain; 2 Department of Public Health, Mental Health and Maternal and Child Health Nursing. Faculty of Nursing, University of Barcelona. Pavelló de Govern, 3Rd Floor, L’Hospitalet de Llobregat, Barcelona, 08907 Spain; 3 Unitat de Suport a la Recerca Metropolitana Sud, Fundació Institut Universitari per a la recerca a l’Atenció Primària de Salut Jordi Gol i Gurina (IDIAPJGol), L’Hospitalet de Llobregat, Barcelona, 08907 Spain; 4 Research Group On Disease, Cardiovascular Risk and Lifestyles in Primary Care, Institut Universitari d’Investigació en Atenció Primària (IDIAP) Jordi Gol, Barcelona, 08007 Spain; 5 Research Group On Environments and Materials for Learning, Universitat de Barcelona, Barcelona, Spain; 6 Fundamental and Clinic Nursing Department, Faculty of Nursing, University of Barcelona, Pavelló de Govern, 3Rd Floor, L’Hospitalet de Llobregat, Barcelona, 08907 Spain; 7 IDIBELL, Bellvitge Biomedical Research Institute, Avinguda de la Gran Via, 199, L’Hospitalet de Llobregat, Barcelona, 08908 Spain; 8 Primary Care Center El Castell, Servei d’Atenció Primària Delta del Llobregat, Atenció Primària Metropolitana Sud, Institut Català de la Salut, Castelldefels, 08860 Barcelona, Spain; 9 Primary Care Center Sant Ildefons. Servei d’Atenció Primària Baix Llobregat Centre, Atenció Primària Metropolitana Sud, Institut Català de la Salut, Cornellà de Llobregat, Barcelona, 08940 Spain; 10 Primary Care Center Montclar and Primary Care Center Camps Blancs. Servei d’Atenció Primària Baix Llobregat Centre, Atenció Primària Metropolitana Sud, Institut Català de la Salut, Sant Boi de Llobregat, Barcelona, 08830 Spain; 11 Chronicity and Complexity Care Unit (UTACC) Baix Llobregat Centre, Atenció Primària Metropolitana Sud, Institut Català de la Salut, Cornellà de Llobregat, 08940 Spain

**Keywords:** Advanced chronic kidney disease, Primary health care, Theory of planned behaviour, Attitudes of health personnel, Qualitative research

## Abstract

**Background:**

Advanced chronic kidney disease (ACKD) is associated with a high risk of adverse cardiovascular and renal events and has a significant impact on quality of life and life expectancy. Several studies have identified areas for improvement in their management in primary care. Some professional and environmental factors can act as key barriers to appropriate care.

**Objective:**

To analyse attitudes, subjective norms, and perceived behavioural control among primary care professionals related to the implementation of an evidence-based approach for individuals with ACKD in primary care.

**Methodology:**

This was a qualitative study using an interpretative phenomenological approach based on the theory of planned behaviour. Two aspects of the evidence-based approach were explored: the implementation of clinical practice guidelines and the utilisation of electronic kidney disease records within the scope of this study. Primary care nurses and physicians participated in a previous pilot interview and five focus groups. Subsequently, a thematic analysis of the gathered data was conducted.

**Findings:**

Thirty-three primary care professionals participated. The emerging themes included: experiences in the management of ACKD (highlighting a distinct profile of older, frail patients with comorbidities masking CKD and a CKD follow-up primarily focused on analytical monitoring and drug adjustment); factors in the professional environment influencing the use of scientific evidence (such as time constraints, excessive electronic health records, and unfamiliar reference guidelines); attitudes towards the application of recommendations on ACKD (recognising limitations of computer systems despite considering them as guidance); and capacities to implement evidence-based recommendations (acknowledging formative needs and challenges in coordinating care with nephrology services).

**Conclusions:**

Several psychological elements identified through the TBP hinder the adequate implementation of an evidence-based approach for individuals with CKD. Attitudes have been identified as factors modulating the use of standardised electronic records. Instead, subjective norms (influences from the professional environment) and perceived behavioral control (perception of capabilities) acted as barriers to the proper application of clinical practice guidelines and standardised records.

**Implications for practice:**

Strategies aimed at optimising the management of people with ACKD should focus not only on training but also on improving attitudes, organisational structures, IT systems and coordination between primary care and nephrology.

**Supplementary Information:**

The online version contains supplementary material available at 10.1186/s12875-024-02418-0.

## Background

Chronic kidney disease (CKD) is a major public health problem. The global prevalence of CKD is estimated to be 13% [1]. Advanced chronic kidney disease (ACKD) comprises individuals with an estimated glomerular filtration rate less than 30 ml/min/1.63 m2 and includes stages G4 and G5 [2]. It accounts for < 5% of all CKD cases but is associated with a very high risk of adverse cardiovascular and renal events and has a significant impact on quality of life and life expectancy [1, 3]. The global mission of the Kidney Disease Improving Global Outcomes (KDIGO) Initiative is to improve the care and health outcomes of people with CKD by promoting coordination, collaboration, and integration of initiatives, while the goals of the International Society of Nephrology are to raise awareness, promote preventive measures, educate professionals in CKD screening, and reduce risk [4]. These guidelines are the framework of care used by local nephrology societies to promote clinical recommendations to patients in primary care [5–7]. Currently, the management of ACKD is predominantly performed in the hospital setting. The prevalence of CKD is increasing in association with obesity, diabetes mellitus and aging [8], and consequently, the number of patients with ACKD will also increase. ACKD is frequently associated with high comorbidity, complexity, and frailty, and approximately one-third of ACKD patients who reach the G5 stage in primary care are not treated with renal replacement therapy [9]. This percentage is even greater for older individuals. Therefore, the expected increase in the number of ACKD patients will require greater involvement of primary care in its management [8]. Primary care professionals are in a privileged position to provide care from the earliest to the most advanced stages, especially in the group of elderly people without renal replacement therapy. However, the primary care approach still has room for improvement [7, 10–13]. CKD management, including diagnosis, prognosis evaluation, monitoring, and risk factor control, can improve [14]. Although the prevalence of CKD is considerable, a large percentage of people with CKD who have improved are unaware that they have CKD, possibly due to a lack of awareness and limited capacity of primary care professionals to adequately identify and treat people with CKD [15].

Context-specific implementation strategies are necessary to optimise the utilisation of scientific evidence. Moreover, research highlights the need to develop standardised care programmes to improve the quality of care for people with ACKD. Indeed, integration into a model comparable to that of people with other diseases would bring similar benefits [10]. Standardised follow-up programs could support the clinical practice of primary care professionals [12] and enhance their evidence base [16]. However, the literature suggests that standardised records are not widely used [17], mainly because of barriers in the care setting [18, 19]. The attitudes of professionals play a key role in the development of actions and therefore in the use of this evidence [20]. However, the implementation of clinical practice guideline recommendations and standardised monitoring systems is a complex process that goes beyond the attitudes of professionals [21]. Some scholars view a lack of knowledge and skills or organisational factors as barriers to implementation [22]. In fact, the Global Kidney Health Atlas notes that some of the barriers to achieving optimal kidney care include factors related to knowledge, attitudes, professional environment factors, and low disease awareness [23]. As such, knowledge, skills, and aspects of work organisation could be seen as shapers of the social norms and perceived behavioural control described by some psychological theories, such as the theory of planned behaviour (TPB) [20]. The TPB is among the most suitable for elucidating and forecasting human behaviour because it pertains to decision-making. According to this theory, behaviour is shaped by a behavioural intention, which, in turn, is influenced by an individual’s attitude towards the behaviour, subjective norms, and perceived behavioural control. Attitude refers to the evaluative belief regarding the outcomes of engaging in a particular behaviour. Subjective norms represent the social pressure to conform to a specific course of action, while behavioural control encompasses one's capability to execute the perceived behaviour, influenced by preidentified obstacles and impediments [24]. As a general guideline, the more favourable the attitude and subjective norm are, and the greater the perceived behavioural control is, the stronger an individual's inclination to enact the contemplated behaviour [20].

Following this line of argument, to gain a deeper understanding of the factors that condition the evidence-based management of people with ACKD in primary care, we performed a qualitative study to explore the psychosocial elements that modulate such management according to the TPB. The aim of this study was to specifically analyse the attitudes, subjective norms, and perceived behavioural control of primary care professionals in managing individuals with ACKD within the primary care setting.

## Methodology

### General description

This qualitative study was part of an exploratory mixed-methods study that will form the basis for the implementation of interventions to improve the management of people with ACKD in the Atenció Primària Metropolitana Sud, a primary care setting south of Barcelona. This area provides care to 1,370,709 people and has 9,196 professionals working in 61 primary care centres.

We used an interpretative phenomenological approach [25, 26] in which experiences are investigated from the perspective of the individual [27]. Focus group accounts were collected from professionals regarding their attitudes, subjective norms, and behavioural control [20] in the management of people with ACKD. The recommended consolidated criteria for reporting qualitative research were followed [28].

The study was approved by the reference primary care Fundació Institut Universitari per la Recerca a l'Atenció Primària de Salut Jordi Gol i Gurina (IDIAPJGol) Clinical Research Ethics Committee (22/092-P).

### Study participants

Participants were purposively selected from among nurses and physicians in the field. The project was presented at a general management meeting, and subsequently, an email was sent with further information. A meeting was arranged at those primary care centers that agreed to participate, and all professionals were invited to participate. The criteria for homogeneity were nurses and physicians working in the Atenció Primària Metropolitana Sud area. The criteria for heterogeneity included sex, professional profile, and level of clinical experience in the management of people with ACKD. We aimed to include various primary care professionals, including nurses and physicians, working in different capacities related to their experience in managing ACKD. This includes roles such as primary care consultation, chronicity profiles, or case manager nurses.

### Data collection

The data were gathered between 1 October 2022 and 31 April 2023 from distinct focus groups of physicians and nurses and from mixed focus groups, aiming to acquire more comprehensive, pertinent, and diverse information relevant to the research query. We initially worked with the professional groups separately to extract maximum information regarding the specific interventions within their daily practice, as well as to identify the specific barriers unique to each profession. This approach aligns with phenomenological principles, wherein the central focus of the study is on the phenomena under examination [29, 30]. Furthermore, standardised follow-up is more commonly conducted by nurses, while physicians tend to rely more on clinical practice guidelines. The mixed group aimed for heterogeneity according to discipline but homogeneity in terms of greater expertise in managing people with advanced chronic diseases. It consisted of physicians specialising in chronicity and nurses specialising in case management. These professionals dedicate more time to treating this profile of patients, and here, the aim was to complement experiences and opinions through the exchange of interventions and barriers and facilitators. We used a script outlining thematic areas derived from the theoretical constructs of the TBP to explore attitudes, subjective norms, and perceived behavioural control regarding the management of individuals with ACKD, as well as the implementation of evidence-based guidelines and standardised electronic records (Additional file 1). Narratives (EPO, ILG, VMF, and MJI) were audio-recorded and subsequently transcribed. Field notes were used during and after the interviews. The interviews, conducted either in Catalan or Spanish depending on the interviewee's preferred language at the time, were transcribed in their respective languages and subsequently translated by a certified translation company. The authors subsequently compared these translations to ensure the semantic accuracy of each phrase. The data were anonymised by assigning them a numerical code.

### Data analysis

The interview data were analysed using thematic analysis procedures [31].

Initially, to obtain a condensed view of the information, the raw data were transformed into usable data by breaking down texts and establishing units of semantic meaning. Relevant data pertaining to the research question were systematically coded. Once all the data had been encoded and the entire dataset had been coded, the codes were grouped into potential subthemes. These subthemes were then integrated within the main themes identified in accordance with the TBP [20]. Subsequently, a review was conducted to assess the coherence of the themes with the coded extracts, ensuring sufficient and relevant data to demonstrate the prevalence of each theme related to the conditioning factors in the approach and use of scientific evidence on ACKD. This process was facilitated by a thematic map, aiding in understanding the relationships between the main themes, subthemes, and codes while applying criteria for internal homogeneity within each theme and external heterogeneity between themes [32]. Finally, the final themes and subthemes were defined and refined, and the report was prepared for thematic analysis of the data [31]. The coding and analysis were conducted by two researchers (ILG and EPO), who developed the thematic map, codebook, subtopics, and topics. A third researcher (VMF) was consulted during the analysis process to resolve any discrepancies in data interpretation [31]. The average interview duration was 55.3 min. The analysis was conducted concurrently with the interviews and continued until thematic saturation. The transcripts were returned to the participants for correction, and no comments were made. All the transcripts were entered into the qualitative analysis software Atlas.ti Web to assist with the data management and analysis. The authors did not employ generative artificial intelligence or AI-assisted technologies in any phase of this research or in its composition. The authors did not employ generative artificial intelligence or AI-assisted technologies in any phase of this research or in its composition.

### Findings

#### Characteristics of the participants

Thirty-three primary care professionals (7 primary care nurse case managers, 14 primary care nurses, 6 family physicians, and 6 family physicians with expertise in complex chronicity) were interviewed in a pilot interview and 5 focus groups in four urban areas and one rural area until information saturation was reached. The average age and average years of professional experience were 46.8 and 19.6 years, respectively (Table 1). Different codes, subtopics (*n* = 11) and topics (*n* = 4) were obtained (Table 2). The topics were experiences in the management of ACKD (topic 1), factors in the professional environment that influence the use of evidence-based action recommendations (topic 2), attitudes toward the use of recommendations (topic 3), and perceived capacities to implement recommendations (topic 4).

**Table 1:** Characteristics of the participants

**Interview**	**Speaker**	**Age**	**Gender**	**Professional profile**	**Years of experience**	**Area**
G01	G01.3	51	F	Family Physician Exp^a^	20	Urban
	G01.2	30	F	Case Manager Nurse in Primary Care	2	Urban
G02	G02.2	40	F	Case Manager Nurse in Primary Care	17	Urban
	G02.3	45	F	Case Manager Nurse in Primary Care	23	Urban
	G02.4	40	F	Case Manager Nurse in Primary Care	17	Urban
	G02.5	34	F	Case Manager Nurse in Primary Care	13	Urban
	G02.6	44	F	Case Manager Nurse in Primary Care	17	Urban
	G02.7	59	F	Case Manager Nurse in Primary Care	38	Urban
	G02.8	62	M	Case Manager Nurse in Primary Care	42	Urban
	G02.9	33	M	Family Physician Exp^a^	5	Urban
G03	G03.2	50	M	Family Physician Exp^a^	20	Rural
	G03.4	39	F	Family Physician Exp^a^	16	Rural
	G03.3	39	F	Family Physician Exp^a^	13	Rural
	G03.5	53	F	Family Physician Exp^a^	23	Rural
G04	G04.3	45	F	Primary Care Nurse	20	Urban
	G04.4	52	M	Primary Care Nurse	26	Urban
	G04.2	52	F	Primary Care Nurse	9	Urban
	G04.5	30	F	Primary Care Nurse	5	Urban
	G04.6	51	F	Primary Care Nurse	27	Urban
G05	G05.2	54	F	Primary Care Nurse	26	Urban
	G05.3	51	F	Primary Care Nurse	26	Urban
	G05.4	61	F	Primary Care Nurse	29	Urban
	G05.5	38	F	Primary Care Nurse	12	Urban
	G05.6	42	F	Primary Care Nurse	18	Urban
	G05.7	57	M	Primary Care Nurse	27	Urban
	G05.8	39	M	Primary Care Nurse	14	Urban
	G05.9	44	F	Primary Care Nurse	23	Urban
G06	G06.2	53	F	Family physician	22	Urban
	G06.3	51	F	Family physician	21	Urban
	G06.4	48	F	Family physician	17	Urban
	G06.5	59	F	Family physician	28	Urban
	G06.6	27	M	Medical resident	3	Urban
	G06.7	56	F	Family physician	26	Urban

^a^Family physician with expertise in chronicity

**Table 2:** Emerging topics, subtopics and codes

***Topics***	***Subtopics***	***Codes***
Experiences in the management of Advanced Chronic Kidney Disease (ACKD)	Profile of patients with ACKD seen in primary care	*“What I normally see are old, fragile, complex patients.*” *G01.2 Primary Care Nurse* “*These filtration values (*< *30) do not pass through primary care because patients already spend enough time on their disease while on dialysis and have enough time to come for follow-up in primary care.* ”*G02.8 Case Manager Nurse in Primary care"* *“In many cases, it may be our own fault for not having trained it well.* ” *G04.3 Primary Care Nurse* *“They are not aware of advanced kidney disease (…). When they become aware, they make changes, if necessary at that point of the disease, in their lifestyles, diet,…*” *G02.3 Case Manager Nurse in Primary Care*
	Masked disease in primary care	“*However, this condition is not considered important for diabetes, hypertension or other (frequent) pathologies. In addition, it should be.* ” *G01.2. Primary care nurse* “*All of us have experienced the disease less, and therefore probably also convey this message less to patients.* ” *G02.9 Family physician with expertise in chronicity* “*I also thought until now that the (advanced CKD patient) went (only) to hospital. I swear.* ” *G04.6 Primary care nurse* “*I think that in general chronic kidney disease is seen as a consequence of other diseases and rarely occurs as an individual entity.* ” *G02.9 Family hysician with expertise in chronicity* “ *I also believe that on many occasions, with the primary care structure, it is very clear to us that we have to attend to the hypertensive patient, the diabetic patient, the patient such as… And we treat it as such. But we don't treat (the patient with CKD), we don't do a blood test, or we don't provide specialised care if it's just kidney failure. It rarely appears in the consultation if you only have kidney failure. It may be difficult to find.* ” *G04.3. Primary care nurse*
	Clinical practices according to professional profile	“*The patient is the one who has to make decisions, right? It is a disease that will progress, and there will come a time when they will have to make important decisions such as dialysis, whether to do it or not, and I think that sometimes it is difficult to reach this point of view, whether they are primary care professionals, specialists, or the patient him/herself* ” *. G02.7 Case Manager Nurse in Primary Care"*
	Patient contextual factors	“ *I mean, it's very difficult. When you see lists of medications and see the amount of things they can't eat… Well, I don't know, it would be difficult for me”* *. G06.3 Family physician* “*They are chronic patients and they are tired of their illness, so for a while they do it (the change of habits), but then you have to insist because…* ” *G05.3 Primary care nurse*
Factors in the professional environment that influence the use of scientific evidence	Pressure from the care environment	“*They look at you, they take (data) pictures of things that are not important. And people think about it. But instead of looking at important things, after the photo, something else comes up”* *G06.2 Family physician*
	Excessive electronic records on a day-to-day basis	“*The perception I have when we do things like this is that then it is like a mental breakdown of having to fill it all in. And then you lose a little bit of focus, so if you look at it as something structural, it is fine because it will help me to control it, it will not slip away; I will keep an eye on it. But the reality is that there are many more things because that is what it is: heart failure, I don't know what, I don't know how many… so I have the feeling that I lose the sense of direction a little bit between all the little things.”* *G06.3 Family physician* *“Yes, I understand that the objective is that everyone, all patients with advanced kidney disease, receive quality care. But if we look at quality care for this, then I think we fall a little short. Obviously, this should be possible in some way, right? But… medicine and nursing are not data. There is data…”* *G06.2 Family physician*
	Implementation of clinical recommendations: poorly accessible and poorly known CKD clinical practice guidelines	*“There is no clear algorithm that gives you clear instructions.* ” *G04.4 Primary care nurse* “*I don't see any pathway. I don't see anything, I mean, the feeling is that we are here in primary care and we are all out of date, and you are looking for a life with your colleagues because no one from there will come to give you any sessions” G05.8 Primary Care nurse* *“There are many things that we do have, but kidney disease, as far as I know, no, there is none.* ” *G01.2 Case Manager Nurse in Primary Care * “*Yes, I think it is necessary to review and reinforce pathways to work all in the same direction* >> *G05.2. Primary care nurse* “*I think are not so easily accessible.”* *G03.2. Family physician with expertise in chronicity*
Attitudes towards the implementation of the CKD recommendations	Standardised care plans for CKD that provide guidance for practice	“*Sometimes, it's a tool (standardised plans) that, when I've used it and seen it over time, is fantastic because it provides access to a multitude of links that offer a wealth of information. However, the issue arises when this information often doesn't have enough time to be conveyed to the patient. (…) There simply isn't enough time to accomplish all of that. But if you have the time, it's remarkable* >> *. G05.7 Primary Care nurse* *“It would help to learn more about advanced chronic kidney disease”* *G05.3 Primary care nurse*
	Unhelpful standardised electronic care plans	“*I would say that I am now indignant because I think that the clinical assessment is detracting, because that is precisely what I am not interested in seeing (with this type of language). It is the least interesting for a patient with complex or advanced chronicity, it is the least informative for colleagues, and it also takes up space and time that makes no sense at all.* ” *G02.8 Case Manager Nurse in Primary Care * *“If you enter through the standardised care plan, no one else sees it, that is, we see it here in the centre, but not in the hospital* ” *G04.6 Primary care nurse* “*I'm following it but they can't see the follow-up that we do, it's clear, it's as if it wasn't followed at all* ” *G02.5 Case Manager Nurse in Primary Care*
Capacities to implement evidence-based recommendations	Lack of training to implement the recommendations in practice	*“I think that the nephrologist is a specialist far removed from primary care. Others do (face-to-face) consultations, endocrinologists, I don't know, and nephrologists are inaccessible.* ” *G02.4 Case Manager Nurse in Primary Care * *“Kidney failure at the nursing level is hardly followed up, if at all* ” *G02.6 Case Manager Nurse in Primary Care *
	Shared management and counselling in the follow-up of ACKD patients	*“That would make it much easier and it would be much easier to follow these guidelines, because it is much more natural, it would give more consistency to the person's case management and it would be easier and better for them.* ” *G03.3. Family physician with expertise in chronicity* *“There is a lack of communication between the different levels and between the different systems.* ” *G05.9 Primary Care Nurse* *“The advantage we have is that you go to the patient's home and see their environment, which is very different from when the patient goes to the nephrology department with dialysis, you don't see the day-to-day reality and we can see that.* ” *G03.2 Family physician with expertise in chronicity*

### Experiences in the management of ACKD

#### Profile of patients with ACKD seen in primary care

In primary care, the predominant profile of people with ACKD corresponds to elderly individuals with comorbidities. The most frequently mentioned were cardiovascular diseases, diabetes and hypertension. Furthermore, in all the focus groups, the associations between frailty and these comorbidities were highlighted.

*“What I normally see are old, fragile, complex patient*s.” G01.2 Primary Care Nurse

Although less common, another profile of people with ACKD was identified—those who were younger and without frailty—who were mainly followed by hospital nephrology and were more disengaged from primary care for this reason.

*“These filtration values (*< *30) do not pass through primary care because patients already spend enough time on their disease while on dialysis and have enough time to come for follow-up in primary care.” * *G02.8 Case Manager Nurse in Primary Care *

A feature repeatedly mentioned is that people most commonly seen in primary care tend to have limited knowledge of their ACKD diagnosis, as opposed to those who require renal replacement therapy.

*“In many cases, it may be our own fault for not having trained it well*.” G04.3 Primary care nurse.

*“They are not aware of advanced kidney disease (…). When they become aware, they make changes, if necessary at that point of the disease, in their lifestyles, diet,…*” *G02.3  Case Manager Nurse in Primary Care.*

#### Masked disease in primary care

In three of the five focus groups, we found stories highlighting that ACKD, both in its diagnosis and in its management in primary care, was masked by other diseases whose follow-up was prioritised over CKD.

“*However, this condition is not considered important for diabetes, hypertension or other (frequent) pathologies. In addition, it should be*.” G01.2. Primary care nurse.

Therefore, the first step is to become aware of the disease they present, both professionals and patients and their families.

“*All of us have experienced the disease less, and therefore probably also convey this message less to patients.*” G02.9 Family physician with expertise in chronicity.

“*I also thought until now that the (ACKD patient) went (only) to the hospital. I swear.”* G04.6 Primary care nurse.

The presence of comorbidities could play a dual role, acting as both a barrier and a facilitator.

On the one hand, three focus groups indicated that when there were numerous comorbidities, they prioritised other diseases over ACKD, which proved to be an obstacle. On the other hand, at times, it serves as a facilitator, as the disease or its progression is frequently detected during the follow-up of other comorbidities. The need to involve nursing input in the proactive follow-up of this population is met.

“*I think that in general, chronic kidney disease is seen as a consequence of other diseases and rarely occurs as an individual entity.*” G02.9 Family physician with expertise in chronicity.

“*I also believe that on many occasions, with the primary care structure, it is very clear to us that we have to attend to the hypertensive patient, the diabetic patient, the patient such as…and we treat it as such. However, we do not treat (for patients with CKD), we do not perform a blood test, or we do not provide specialised care if it is just kidney failure. It rarely appears in the consultation if you only have kidney failure. It may be difficult to find.”*  G04.3. Primary care nurse.

#### Clinical practices according to professional profile

In relation to the management of ACKD, the most important elements identified included the control of cardiovascular risk factors, regular blood and urine test follow-up, review and adjustment of prescribed medication, management of dietary habits and controls, social support and, in the final stages, decision making to ‘stop doing’, i.e., to reduce medical interventions and prioritise quality of life. However, the implementation barriers discussed throughout the study were also reported.

In terms of the nursing approach, health education predominated over lifestyle modification, suggesting on several occasions the importance of a low-sodium diet and strict water restriction, as well as interventions to measure anthropometric variables, vital signs and medication review (in all nursing focus groups). Caregiver support and intervention in the socioeconomic dimension also appeared, in contrast to the findings for the groups treated by physicians only. In terms of the approach taken by primary care physicians, in all of the focus groups reported, the main focus was on the review of nephrotoxic drugs, follow-up tests and the detection of complications. In contrast, in more specialised chronicity roles (G03.2, G03.5, G02.3, G02.6, and G02.7), such as case managers and chronicity physicians, aspects such as shared decision making, anticipation of possible complications arising from disease progression and 'stop doing' interventions predominated.

*“The patient is the one who has to make decisions, right? It is a disease that will progress, and there will come a time when they will have to make important decisions such as dialysis, whether to do it or not, and I think that sometimes it is difficult to reach this point of view, whether they are primary care professionals, specialists, or the patient him/herself* ” *. G02.7 Case Manager Nurse in Primary Care.*

#### Patient contextual factors

The common presence of multiple comorbidities often poses a challenge for professionals, hindering adherence to recommendations regarding medication, diet, and other lifestyle factors. For example, long lists of medicines can trigger adverse effects and poor adherence. In addition, older age, according to the perceptions of professionals, is a barrier to lifestyle change. Another difficulty that was strongly emphasised was the socioeconomic conditions that are undermined by the increased overall frailty of people with ACKD. These findings were evident in all the focus groups.

“*I mean, it is very difficult. When you see lists of medications and see the amount of things they cannot eat… Well, I do not know, it would be difficult for me.*” G06.3 Family physician.

“*They are chronic patients, and they are tired of their illness, so for a while they do it (the change of habits), but then you have to insist because…”* G05.3 Primary care nurse.

### Factors in the professional environment that influence the use of scientific evidence

#### Pressure from the care environment

Limited time was identified as the primary barrier to seeking and implementing the recommended guidelines. Some professionals across four focus groups addressed this challenge by implementing time optimisation strategies and fostering teamwork. Specifically, physicians employed a wider range of strategies regarding clinical guidelines (e.g., consulting with colleagues G01.3 by storing reference guides in folders readily accessible for daily practice G03.5), while nurses concentrated on standardised electronic records (prioritising the disease with the poorest control, G05.8). Both groups observed that collaborative working helped alleviate pressure barriers in the work environment.

Another obstacle to the utilisation of specific CKD systematic records lies in the annual incentives for accessing general electronic records. These incentives encourage all primary care professionals to record other patient clinical variables that are not specific to ACKD. This resulted in family physicians (G01.3, G06.2, G06.3, G06,5) prioritising the recording of those nonspecific ACKD variables within a limited timeframe rather than the typical standardised monitoring variables that should be applied to CKD patients. Consequently, professionals reported recording variables that were not the most crucial and thus failed to deliver the care considered a priority.

“*They look at you; they take (data) pictures of things that are not important. (…) But, instead of focusing on significant matters, something different arises after taking the photo.*” G06.2 Family physician.

#### Excessive electronic records on a day-to-day basis

In the nursing focus groups (G04, G05, and the nurse case managers from G02), standardised care plans were seen to create surplus documentation when caring for individuals with multiple conditions, as each plan is tailored to address specific needs. This means that the clinical care of the individual requires the implementation of several plans, multiplying the records. This aspect was identified as a barrier to implementation. In fact, nurses who try to implement the care plan together with other plans for other diseases describe the recording situation as complicated, attributed to its terms such as *"surviving" (G05.8)* or *"juggling" (G05.9)*, due to the limited time available during the consultation with the patient.

 “*The perception I have when we do things like this is that then it is like a mental breakdown of having to fill it all in. In addition, then you lose a little bit of focus, so if you look at it as something structural, it is fine because it will help me to control it, it will not slip away; I will keep an eye on it. However, the reality is that there are many more things because that is what it is: heart failure, I do not know what, I don't know how many… so I have the feeling that I lose the sense of direction a little bit between all the little things.*” G06.3 Family physician.

 “*Yes, I understand that the objective is that everyone, all patients with advanced kidney disease receive quality care. But if we look at quality care for this, then I think we fall a little short. Obviously, this should be possible in some way, right? But… medicine and nursing are not data. There is data…*” G06.2 Family physician.

#### Implementation of clinical recommendations: poorly accessible and poorly known CKD clinical practice guidelines

It was often considered that there is more knowledge of other diseases that are more prevalent in primary care with respect to the reference guidelines of the hospital and primary care settings; therefore, they are applied with a certain cohesion between professionals in both settings. However, in relation to CKD and, specifically, ACKD, none of the participants in the study clearly identified the reference guidelines. Although some professionals were aware of some of the recommendations, they doubted that they were the same as those given by the reference nephrology services and therefore doubted whether a unified message was being given from the two areas.

“*There is no clear algorithm that gives you clear instructions.*” G04.4 Primary care nurse.

*“I don't see any pathway. I don't see anything, I mean, the feeling is that we are here in primary care and we are all out of date, and you are looking for a life with your colleagues because no one from there will come to give you any sessions*” G05.8 Primary Care nurse.

“*There are many things that we do have, but kidney disease, as far as I know, no, there is none.*” G01.2 Primary care nurse case manager

As a proposal to improve this aspect, they reported that territorial care processes could contribute to improving and updating practices through recommendations in the guidelines (focus groups G03, G05, G06). This approach would make it easier to work in a more unified way at the territorial level, i.e., in primary and hospital care settings.

“*Yes, I think it is necessary to review and reinforce pathways to work all in the same direction”* G05.2. Primary care nurse.

The lack of updating of care pathways, according to professionals, has contributed to the dilution of guidelines and pathways over time, resulting in a lack of knowledge about them. Integration into the computerised medical records platform was advocated, as had been done in the past for other pathologies. In a primary care context with significant variability in the reasons for consultation and care pressure, they considered that it is necessary to activate digital resources that facilitate access to the best available evidence in a simple way.

“*I think are not so easily accessible”* G03.2. Family physician with expertise in chronicity.

### Attitudes toward the implementation of the CKD recommendations

#### Standardised care plans for CKD that provide guidance for practice

Some participating nurses highlighted that the use of these standardised electronic records in primary care carries controversial implications within the same team. While some nurses perceive them as burdensome and disconnected from day-to-day usefulness (G02.8, G04.6, G06.2), others view them as practical guidelines that establish the foundation for standardised care among professionals who utilise them, aiding in enhancing knowledge about the disease and its management (G01.2, G02.6, G04.5, G05.3, G06.5).

“*Sometimes, it's a tool (standardised plans) that, when I've used it and seen it over time, is fantastic because it provides access to a multitude of links that offer a wealth of information. However, the issue arises when this information often doesn’t have enough time to be conveyed to the patient. (…) There simply isn't enough time to accomplish all of that. But if you have the time, it's remarkable * .”G05.7 Primary Care nurse.

*“It would help to learn more about advanced chronic kidney disease” *G05.3 Primary care nurse.

#### Unhelpful standardised electronic care plans

There was an attitudinal barrier related to the belief that standardised care plans were not useful for sharing health information among colleagues. The professionals argued for two technical reasons. First, this information tended to be met with resistance from professionals who use and review it (G03,2, G02.8), primarily because of its format within the patient's medical records. It is often described as lacking clarity or personalisation to the patient's specific circumstances (G02,3, G02,7, G02.8).

“*I would say that I am now indignant because I think that the clinical assessment is detracting, because that is precisely what I am not interested in seeing (with this type of language). It is the least interesting for a patient with complex or advanced chronicity, it is the least informative for colleagues, and it also takes up space and time that makes no sense at all.”* G02.8 Primary care nurse case manager.

The second reason they perceived standardised electronic records as being of little use was because, at present, in the shared medical records—accessed electronically from other healthcare settings and providers, such as hospitals—only some clinical follow-up data from primary care is visible, not all, as is the case with electronic records for conditions such as CKD. These are only accessible in the primary care setting and not in other hospital settings (G04,6, G02.4, G02.5, G02.6). Therefore, healthcare professionals opt to use alternative clinical records that are visible in shared medical records, even though they are not standardised electronic records for CKD patients. Knowing that important follow-up data entered into electronic CKD records will not be seen by other hospital colleagues who are involved in the care of these patients, such as nephrologists, leads professionals to choose other types of clinical records. This approach is relevant because, on many occasions, clinical information of interest to both parties is shared.

*“*If you enter through the standardised care plan, no one else sees it, that is, we see it *here* in the centre, but not in the hospital*” *G04.6 Primary care nurse

*“I’m following it but they cannot see the follow-up that we do, it is clear, it is as if it was not followed at all” *G02.5 Case Manager Nurse in Primary Care.

### Capacities to implement evidence-based recommendations

#### Lack of training to implement the recommendations in practice

Practitioners reported that the CKD care plan improves safety and guides practice. However, to optimise its use, they underlined the need to implement specific training strategies to enable its correct application. Team sessions on various clinical management topics are recognised as an important element in keeping professionals up to date and cohesive in the management of the population with chronic health conditions. However, in line with the masking of CKD with other diseases, this dynamic is also reflected in primary care team sessions (G02.3, G06.4) and nonexisting face-to-face consultations with nephrology professionals in the hospital setting (G03.2, G03.5), considering that nephrology services are distant from primary care (G02.4, G02.9, G06.3).

*“I think that the nephrologist is a specialist far removed from primary care. Others do (face-to-face) consultations, endocrinologists, I don't know, and nephrologists are inaccessible.*” *G02.4 Case Manager Nurse in Primary Care.*

Another aspect related to the capacity for a specific approach to this disease is that, thus far, the primary care nurse has not been fully and proactively engaged in the overall follow-up of this patient profile. This lack of involvement does not allow for the teamwork that is essential for jointly addressing ACKD, as indicated in the recommendations.

“*Kidney failure at the nursing level is hardly followed up, if at all*” G02.6 Case Manager Nurse in Primary Care

#### Shared management and counselling in the follow-up of ACKD patients

A frequently encountered situation identified was the lack of professional meetings with specialists from the referral service, as well as the lack of two-way communication channels with them. This aspect, which was repeatedly mentioned in the focus groups, has been considered an important factor associated with the implementation of the recommendations. The potential to exchange viewpoints and treatments is considered crucial, as it enhances understanding and boosts confidence in their application. Professionals emphasised that collaborative follow-up (G01.2, G03.3), based on the same evidence-backed guidelines, would contribute to enhancing care for this particular group.

“*That would make it much easier? to follow these guidelines, because it is much more natural, it would give more consistency to the person's case management and it would be easier and better for them.*” *G03.3. Family physician with expertise in chronicity.*

“*There is a lack of communication between the different levels and between the different systems.*” G05.9 Primary Care Nurse

Virtual consultations have become the standard method for discussing clinical management between primary care providers and hospitals. However, they are one-sided, which hinders the effective exchange of information among professionals in both settings (G06,2, G06.3, G06.4, G03.2, G03.4, G03.5). Professionals advocated for interactions that create opportunities for interdisciplinary and transdisciplinary care (G01.2, G03.3, G04.2, G04.3). While primary care providers seek guidance from the nephrology department, professionals also highlight that primary care providers possess a deeper understanding of the patient's sociofamilial context. Hence, deferred teleconsultation does not entirely resolve this issue, as collaborative work between both services is necessary. Participants reported that this collaboration should consider all perspectives to enable genuine shared decision-making.

“*The advantage we have is that you go to the patient's home and see their environment, which is very different from when the patient goes to the nephrology department with dialysis; you don't see the day-to-day reality and we can see that.*” G03.2 Family physician with expertise in chronicity.

## Discussion

In this qualitative study, we specifically identified attitudinal, environmental, and behavioural control elements as outlined in the Theory of Planned Behaviour concerning ACKD management within a population of primary care nurses and physicians.

Although qualitative studies on practitioners' views on the management of ACKD exist [12], to our knowledge, this is the first study to use the conceptual components of this theory to study factors associated with practitioners' implementation of practice-based management of ACKD. To contextualise the elements of the theory studied, we investigated the characteristics of the people with ACKD most frequently seen in primary care from the perspective of professionals. First, the usual profile is that of an elderly person with global frailty and comorbidities. These findings are supported by the literature, in which CKD has strong links with chronic diseases [2, 33], and their accumulation occurs with age and leads to frailty [34]. In the present study, the context of comorbidity and its consequences was recognised as a barrier to the application of evidence-based clinical recommendations. Furthermore, Squires et al. [35] In a 2019 study on contextual attributes for practitioners' use of evidence, patient context was one of the most frequently cited attributes more than 90% of the time [35]. Indeed, Kim et al. [36] reported that uncertainty and social support (which were also identified in our study population) were important factors associated with adherence in CKD patients [36]. Another barrier identified in the present study was educational barriers in terms of patients’ lack of knowledge about their disease and even lack of disease awareness. This finding has also been echoed in other studies, which estimate that 90% of kidney patients are unaware of their diagnosis [15]. In our study, professionals acknowledged that they do not play a prominent role in informing patients about this pathology, a situation that has also been observed in other primary care settings related to CKD [37]. Indeed, a systematic review on barriers to and facilitators of CKD treatment in primary care highlighted a deficiency in resources for patient education [38]. With regard to the approach to treating ACKD in primary care, another relevant finding of the study was that this disease is neglected in relation to other active pathologies, as the interventions recommended in the clinical practice guidelines were not given the same weight. The causes were diverse and included issues such as time constraints, limited accessibility, lack of familiarity with reference clinical guidelines, and inadequate professional training. In this regard, the results were consistent with multiple studies that have shown CKD to be a significant clinical problem with lower priority [38]. Additionally, a worldwide study revealed that professional barriers, such as low knowledge, negative attitudes, and limited professional awareness, were prevalent in more than 80% of the surveyed countries [39]. One possible explanation for this could be the examination of the perspectives of family physicians involved in CKD care, highlighting issues such as a lack of confidence and limited experience in follow-up care, among other factors [12].

Although several authors have investigated the approach and limitations of family physicians in primary care for patients with ACKD, few studies have explored this aspect in nurses. In our study, most nurses expressed the belief that ACKD was addressed only in the hospital and that it was not proactively and comprehensively followed up in primary care for this reason. This indicated that nurses have not fully developed their contribution to the care of people with ACKD in primary care and that the same standards applied in other pathologies are not used. The work of primary care nurses in the care of people with ACKD requires the systematisation of evidence-based care, as indicated in the healthcare context in which this study was carried out [40]. In addition, our study revealed the need to involve nurses, especially since patients were identified as having little knowledge of self-management of ACKD, which implies significant educational needs. To promote self-care, primary care nurses, as experts in health education, need to include people with ACKD in global and proactive follow-up, as indicated by multidisciplinary models of care [41]. One study highlighted the need to improve the accessibility of educational interventions for patients with ACKD among nephrology nurses [13]. It is therefore reasonable that nurses in this primary care setting should be able to develop educational interventions to optimise patient self-care. Enhancing the role of nurses within the multidisciplinary care model for people with ACKD would contribute to slowing disease progression, decreasing mortality and reducing the annual costs of the disease [41].

To investigate the subjective norms that influence the application of ACKD management guidelines, we examined factors in the professional environment in a universal health primary care setting. The pressure of care was identified at two levels: limited time and the excessive burden of electronic records. Historically, time constraints in patient consultations have been recognised as barriers to the implementation of evidence-based practices [16]. However, it is important to consider the excessive electronic record-keeping demands placed on professionals. In the context of our study, implementing standardised plans and evidence-based clinical recommendations was not an easy task, as the participants assured that it requires updated knowledge and skills for their integration into everyday life [16]. This scenario has prompted a sense of resistance towards electronic records, attributed to the sheer volume that professionals are required to manage when addressing comprehensive patient care, encompassing all their comorbidities. In a systematic review examining barriers to and facilitators of e-health implementation, while mismatch with daily clinical practice was acknowledged, this particular issue was not explicitly pinpointed [42]. This could be due to the desire to integrate all dimensions of health and the profile of patients in primary care. Another subjective standard identified was the accessibility of clinical practice guidelines in the work environment. In our study, this issue was evident in all the focus groups, where reference Clinical Practice Guidelines for CKD were reported to be unfamiliar to practitioners and perceived as inaccessible. Although accessibility has improved dramatically through internet search engines, keeping up to date with the literature, they reported that this improvement was difficult due to the wide variety of studies and information available. Selecting the best evidence and in cohesion with the rest of the providers requires efforts on the part of health care companies to implement these strategies in the context and organisation [43].

In exploring further attitudes toward the application of evidence-based practices, subtopics related to standardised electronic CKD plans emerged. On the one hand, and in line with other studies, these findings can guide practice and contribute to professional knowledge of ACKD. This is because standardised electronic records have the potential to enhance the quality and coordination of care for individuals with multiple chronic diseases [44]. They incorporate recommendations for patient follow-up, with technological support being identified as the most common facilitator [38]. On the other hand, a barrier to their use was that they were considered not very useful for recording and interprofessional communication. This remains a major barrier to the management of CKD [38]. Some research identifies the quality of electronic records as a challenge, and proposals focus on improving the functionality of the software and improving multidisciplinary cooperation [45]. Similarly, in our study, standardised records were also seen as hindering communication and diminishing the recognition of the nurse's role, as they are primarily responsible for implementing this type of documentation within our study’s context.

In exploring the elements of perceived behavioural control specifically, two interrelated subtopics emerged. On the one hand, training to implement the recommendations is lacking. 

This can be explained by three related factors: dissatisfaction with the guidelines to be followed, perceived lack of knowledge, and a lack of awareness of support resources [38]. The present study revealed that the implementation of standardised care plans, in addition to overcoming the aforementioned environmental barriers, must be complemented by specific training strategies within the primary care team. In addition, consulting with nephrology providers could improve this approach to incorporate the recommendations into practice. Finally, aligning with findings from other studies, enhancing the collaborative relationship between primary care and nephrology professionals was suggested as a factor to increase perceived behavioural control [12, 13, 38, 46]. In the context of our study, professionals perceived the necessity of establishing bidirectional communication channels to enable the shared follow-up of people with ACKD.

### Limitations

This study has several limitations. First, professionals who agreed to participate were recruited through the primary care centres. Afterwards, nurses and physicians from the participating centres were summoned and invited to attend on an agreed-upon day and time. To reach a wider audience, the focus groups were conducted in the workplace. However, it is possible that we may have gathered results on the attitudes, subjective norms, and behavioural control of professionals most involved in the care of individuals with this condition, potentially overlooking the barriers faced by those less familiar with the disease. This was an aspect we could not control in the research field. Additionally, another aspect beyond our control was the age of the participants. The studied sample had an average age of 46 years, meaning that we may have omitted elements of the TPB related to younger ages, for instance, concerning the implementation of standardised electronic records. Second, we sought to analyse the three main elements described in the TPB, which are the conditioning factors for following evidence-based recommendations. When guiding research based on this theory, we have not explored other elements that could influence it, such as moral norms, an extension of the TPB, which should be considered to fully understand the psychological factors associated with evidence-based approaches to ACKD. The moral norm is understood as a person's perception of the appropriateness of certain behaviours [24]. The authors of the TPB argue that moral a reliable predictor of behaviour in situations where strong social pressures exist, as in the case of the present study. Therefore, the fact that some beliefs and experiences analysed in this study are linked to social pressure suggests that the values and beliefs studied alongside the moral norm could play a significant role in analysing the use of evidence-based practice. In essence, further investigation is needed to comprehend why key guidelines for treating ERCA have not been implemented, even when they are known or if the environment supports them positively. Perhaps exploring this aspect through moral standards is necessary. Additionally, by examining the three elements of the TPB, we may have overlooked other facets related to the application of the TBP or the context in which it is studied, as indicated by Squires et al. [35] in a study on different contextual attributes that influence the application of evidence-based practice. Finally, our results are based on self-reported practices, and we do not know to what extent self-reported practices and other contextual factors reflect reality and how patients perceive this care. In future research, it would be useful to analyse the influence of the abovementioned aspects that may help to gain a deeper understanding of the factors behind evidence-based practices in the management of people with ACKD.

## Conclusions

The clinical, social, and healthcare context of CKD patients presented challenges in implementing an evidence-based team approach. This approach has impacted the application of clinical practice guidelines and standardised care plans. Several psychological elements identified through the TBP make it challenging to adequately implement an evidence-based approach for people with ACKD. Attitudes have been recognised as factors that modulate the use of standardised electronic records. Professionals suggested enhancing information technology systems and effectively integrating them into shared medical records. However, subjective norms (influences from the professional environment) and perceived behavioural control (perception of capabilities) acted as barriers to the appropriate application of clinical practice guidelines and standardised records. Professionals advocated overcoming these barriers through team clinical sessions, collaborative teamwork involving nurses in active ACKD monitoring, and cooperation with referring nephrology services.

### Implications for practice

This study aimed to specifically understand the attitudes, subjective norms, and behavioural control underlying the use of the evidence-based approach for people with ACKD in primary care. The TBP aided in identifying the psychological elements underlying an evidence-based approach for individuals with ACKD. On the one hand, standardised electronic ACKD records face significant limitations in terms of attitudes, subjective norms, and behavioural monitoring. In the short term, to address this issue, strategies should focus on enhancing positive attitudes, which guide professional practice, and counteracting negative attitudes, thus improving their utility to enhance work and the visibility of interventions. In the long term, improving subjective norms would involve reducing the overall demand for records in primary care, while enhancing perceived behavioural control would involve promoting the use and sharing of records among all team members.

On the other hand, the implementation of CKD clinical practice guidelines identified barriers related to subjective norms and behavioural control. In the short term, strategies to address this issue should aim to integrate these guidelines into workplace information systems so that they are readily accessible and can be shared by colleagues in team sessions, enabling continuous updating. This strategy lays the groundwork for improving perceived behavioural control, with longer-term strategies including the development of new communication channels for advice and shared management of patients between primary care and nephrology professionals.

### Supplementary Information

Supplementary Material 1.

## Data Availability

The datasets used, generated or analyzed during this study are available from corresponding author on reasonable request.
