# Supplementary material for: Exploring the factors influencing evidence-based approaches to advanced chronic kidney disease: a qualitative study involving nurses and physicians
Source: BMC Prim Care. 2024 May 21;25:177. doi: 10.1186/s12875-024-02418-0 (PMC11107048; doi:10.1186/s12875-024-02418-0)
Supplement: Supplementary file 1 — Supplementary Material 1. [file 12875_2024_2418_MOESM1_ESM.docx]

**FOCUS GROUP CONTENT SCRIPT FOR PRIMARY CARE PROFESSIONALS. MARECA STUDY**

| ***We would like to hear your opinions and experiences about:*** | |
| --- | --- |
| **Advanced chronic kidney disease** | *1 How would you describe the profile of the person with ACKD?* |
|  | *2 What aspects have you found to be the most important in working with ACKD patients?* |
| **Practices of primary care professionals for ACKD** | *3 Could you describe what your usual practice is with people with ACKD? What specific treatment recommendations do you give to people with ACKD?* |
|  | *4 What do you think are the main difficulties that ACKD patients have in following the prescribed therapeutic recommendations?* |
| **Use of scientific evidence in the management of ACKD** | *5 Do you use ACKD specific handling guides? Which ones?* |
| **Attitudes toward the use of evidence**  It is the degree to which a person has a favourable or unfavourable evaluation regarding adopting a behaviour | *6 Do you use AI tracking variables for this disease? Do you know/use ARES tracking? (in general and for CKD?)* |
|  | *7 What do you think of a structured monitoring system specific to the disease? (analytical protocol, ares...)*  *Do you consider the structured follow-up to the Active intelligence (AI) /ARES in chronic kidney disease to be facilitating and convenient or hindering and inconvenient? And do you consider it realistic? What would it help you with?* |
|  | *8 What difficulties do you encounter in providing appropriate/evidence-based follow-up for people with ACKD?* |
|  | *9 What benefits do you think comes from consulting the clinical guidelines for the management of ACKD? And the benefits of AI or ARES support?* |
| **Subjective norms**  It is the perceived social influence to perform the behaviour or not. In this context, it is considered that the working environment of the professionals would confirm this factor | *10 Do you consider that the ACKD guidelines and protocols are accessible in your day-to-day life?* |
|  | *11 Which factors of the professional environment (team organisation, computer level) do you think help you or don't help you use them? For example, regular sessions/meetings with referring hospital Nephrology members?* |
|  | *12 What proposals do you think would be needed to improve the use of protocols or structured monitoring (ARES or Active Intelligence)?* |
|  | *13 What would you expect from the relationship with a referral on the nephrology team and/or with the nephrology service?* |
| **Perceived behavioural control**  It is the ability to perform the perceived behavior (PBE), and this is determined by the obstacles and impediments found in advance | *14 What strengths would you say you have, each of you, to consult and apply the recommendations of the guides? What I think I do well in practice, I apply these criteria, I am good at consulting and applying the guides...* |
|  | *15 What do you think you would need to improve your use of evidence/guidelines or to use structured monitoring?*  *Structured monitoring brings you the summary of the evidence on the AI screen. Do you think this is a help?* |
|  | *16 What aspects of the care environment determine your* capacity *to apply the guides? Both positive and negative. What capabilities do you have to reverse them?* |
